# CD73-Mediated Formation of Extracellular Adenosine Is Responsible for Adenosine A_2A_ Receptor-Mediated Control of Fear Memory and Amygdala Plasticity

**DOI:** 10.3390/ijms232112826

**Published:** 2022-10-24

**Authors:** Ana Patrícia Simões, Francisco Q. Gonçalves, Daniel Rial, Samira G. Ferreira, João Pedro Lopes, Paula M. Canas, Rodrigo A. Cunha

**Affiliations:** 1 CNC-Center for Neuroscience and Cell Biology, University of Coimbra, 3004-504 Coimbra, Portugal; 2 Faculty of Medicine, University of Coimbra, 3004-504 Coimbra, Portugal

**Keywords:** CD73, ecto-nucleotidases, adenosine, A_2A_ receptors, fear memory, synaptic plasticity, LTP, P2 receptors

## Abstract

Adenosine A_2A_ receptors (A_2A_R) control fear memory and the underlying processes of synaptic plasticity in the amygdala. In other brain regions, A_2A_R activation is ensured by ATP-derived extracellular adenosine formed by ecto-5′-nucleotidase or CD73. We now tested whether CD73 is also responsible to provide for the activation of A_2A_R in controlling fear memory and amygdala long-term potentiation (LTP). The bilateral intracerebroventricular injection of the CD73 inhibitor αβ-methylene ADP (AOPCP, 1 nmol/ventricle/day) phenocopied the effect of the A_2A_R blockade by decreasing the expression of fear memory, an effect disappearing in CD73-knockout (KO) mice and in forebrain neuronal A_2A_R-KO mice. In the presence of PPADS (20 μM) to eliminate any modification of ATP/ADP-mediated P2 receptor effects, both AOPCP (100 μM) and the A_2A_R antagonist, SCH58261 (50 nM), decreased LTP magnitude in synapses of projection from the external capsula into the lateral amygdala, an effect eliminated in slices from both forebrain neuronal A_2A_R-KO mice and CD73-KO mice. These data indicate a key role of CD73 in the process of A_2A_R-mediated control of fear memory and underlying synaptic plasticity processes in the amygdala, paving the way to envisage CD73 as a new therapeutic target to interfere with abnormal fear-like emotional processing.

## 1. Introduction

The function of the amygdala is tightly associated with the encoding of emotional experiences [1,2]. Accordingly, the amygdala has a central role in processing fear-related associative processes which are encoded through adaptive synaptic efficiency, namely, long-term potentiation (LTP), in synapses of the basolateral amygdala receiving cortico-thalamic sensory information [1,2]. Several modulators are known to fine-tune amygdala LTP to influence fear memories, and detailing their function is important to devise novel strategies to interfere with emotional dysfunction and phobia, which are major burdens of disease in Western countries [3].

Adenosine A_2A_ receptors (A_2A_R) is one such neuromodulation system that controls the expression of associative fear memories [4,5] as well as emotional dysfunction [6,7,8,9,10]. The relevance of A_2A_R in the control of emotional dysfunction is further heralded by the association of polymorphisms of the ADORA2A gene with panic disorders [11,12], anxiety (e.g., [13]) and depression [14]. A_2A_R are present in the amygdala [15,16], mainly in synaptic contacts [5], where their activation has been reported to control the excitability of principal neurons [17], the strength of the inhibitory network [18] and the magnitude of LTP in excitatory synapses [5], in accordance with the key role of the A_2A_R transducing system (protein kinase A and CREB; reviewed in [19]) to control amygdala LTP (e.g., [20,21]). A_2A_R are mostly dedicated to controlling synaptic plasticity in different brain regions [22], and their activation is ensured by a particular pool of adenosine formed by ecto-nucleotidases [23,24,25,26] coupled to a larger ATP release upon greater frequency or more intense stimulation [27,28]. Importantly, the overactivation of A_2A_R is associated with the onset of brain dysfunction (reviewed in [29]), namely of emotional disturbances, as best heralded by the ability of A_2A_R antagonists to attenuate mood dysfunction [7,8,10]. However, it has not yet been tested if the blockade of ecto-5′-nucleotidase or CD73, the rate-limiting ecto-nucleotidase controlling ATP-derived adenosine formation [30,31], is responsible for the engagement of amygdala A_2A_R in controlling fear memory processing, as it is observed in the hippocampus to control spatial memory deficits in animal models of Alzheimer’s disease [25] or in the striatum to control motor dysfunction in animal models of Parkinson’s disease [32]. Therefore, we have now tested whether a previously validated inhibitor of CD73 (α,β-methylene ADP, AOPCP) could modify fear memory processing and synaptic plasticity in the amygdala in an A_2A_R-dependent manner.

## 2. Results

### 2.1. Inhibition of CD73 Attenuates Fear Memory

**Figure 1:**
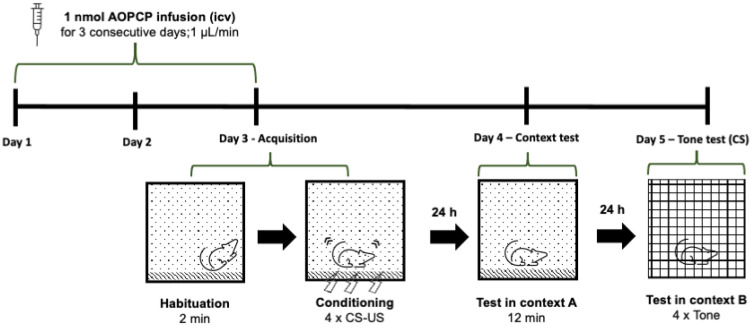
Schematic presentation of the fear conditioning protocol. AOPCP (1 nmol per ventricle) or vehicle were infused into both lateral ventricles (icv) of mice at a rate of 1 µL/min, once a day, throughout 3 consecutive days. On day 3, 1 h after icv infusion and after a habitation period of 2 min in the conditioning chamber (context A), mice were cued-fear conditioned (a tone (CS) of 80 dB was presented during 20 s and terminated with a footshock (US) of 0.5 mA and 2 s duration; 4 CS–US pairings were presented at 140 s intervals). On day 4, 24 h after fear conditioning, mice were re-exposed to context A during 12 min to probe for contextual fear memory. On day 5, cued-fear memory was tested in a different context (context B) for 12 min by presenting the tone 4 times at 140 s intervals after a 2 min habituation period.

**Figure 2:**
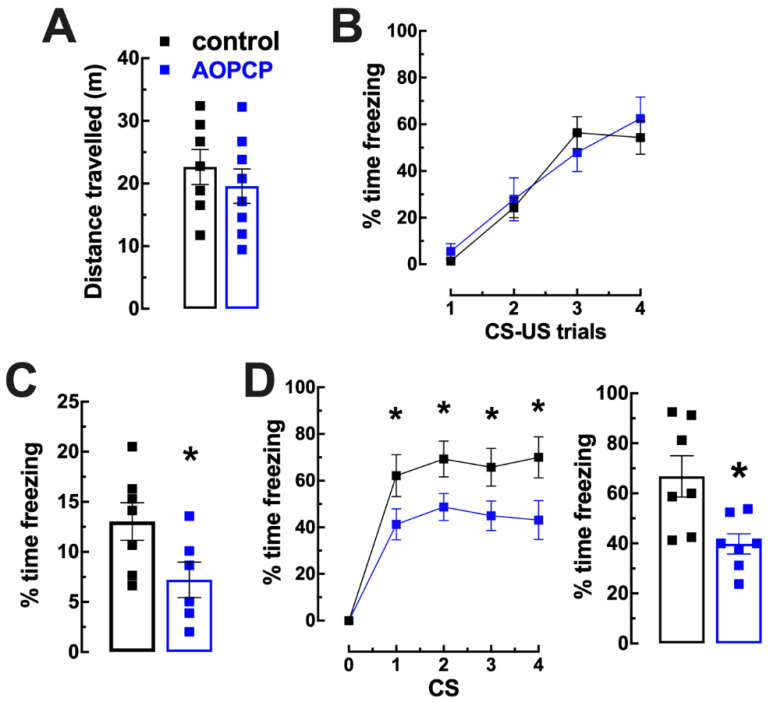
Pharmacological inhibition of CD73 before fear conditioning decreased contextual and cued fear memory in wild type mice. (**A**) Locomotor activity of AOPCP- and saline-treated wild type mice. The icv administration of AOPCP to CD73 wild-type (WT) mice (WT littermates of the CD73 knockout colony) did not change the locomotor profile compared to the control (saline-treated) group. (**B**) Fear conditioning of AOPCP-treated and saline-treated mice: freezing responses to four repeated presentations of a tone (CS- 20 s of white noise, 80 dB) paired with a footshock (US- 2 s, 0.5 mA footshock) were similar between both groups of mice, showing a similar learning curve. (**C**) Contextual fear memory: AOPCP-treated mice displayed a lower percentage of freezing behavior comparing to the control group upon re-exposure to the conditioning chamber (context A) in the absence of the US, 1 day after fear conditioning. (**D**) Cued fear memory: AOPCP-treated mice displayed a lower freezing behavior compared to the control group upon each presentation of the CS (4 trials after 2 min habituation (H) period) in the absence of the US, in a novel context B, 2 days after fear conditioning (graph on the **left**) and an overall lower percentage of freezing throughout the experiment (graph on the **right**). The values are mean ± SEM of n = 7–9 experiments; * *p* < 0.05, unpaired Student’s *t* test.

We previously described that mice injected with AOPCP display a similar pattern of spontaneous locomotion in the open field compared to control mice [24] as well as a similar physical performance at low to moderate intensities [33] and a similar spatial reference memory performance [25]. The impact of AOPCP, applied intracerebroventricularly, was now tested in fear conditioning memory (Figure 1). We first confirmed that AOPCP-treated mice had a similar spontaneous locomotion profile in the open field (Figure 2A). During fear conditioning, mice treated with vehicle or with AOPCP displayed a similar increased freezing with successive CS–US pairings (F_3,42_ = 0.952, *p* = 0.424; Figure 2B). This observation that the acquisition of fear was similar in both groups of mice suggests that the inhibition of CD73 did not affect shock perception, responsiveness or formation of the CS–US association. One day after fear conditioning, saline-treated mice froze 13.03 ± 1.89% (n = 7) of the total time (12 min) of re-exposure to the conditioning chamber A (Figure 2C); this was attenuated by AOPCP (n = 9) to 5.81 ± 2.61% of the total time freezing (t_11_ = 2.227 *p* = 0.048, two tailed unpaired *t*-test; Figure 2C). A similar pattern was observed when mice were placed in a novel context B 2 days after fear conditioning (day 3) and re-exposed to the same CS (Figure 2D): control mice increased their freezing upon presentation of the CS (n = 7) (Figure 2D), and this was inhibited by AOPCP (n = 7) (CS_1_: saline 62.14 ± 8.98%, AOPCP 41.25 ± 6.66%, *p* = 0.03; CS_2_: saline 69.29 ± 7.67%, AOPCP 48.75 ± 5.81%, *p* = 0.03; CS_3_: saline 65.71 ± 8.12%, AOPCP 45.00 ± 6.34%, *p* = 0.03; CS_4_: saline 70.00 ± 8.80%, AOPCP 43.13 ± 8.34%, *p* = 0.007), as demonstrated by a two-way ANOVA interaction (F_4,52_ = 2.87, *p* = 0.032).

**Figure 3:**
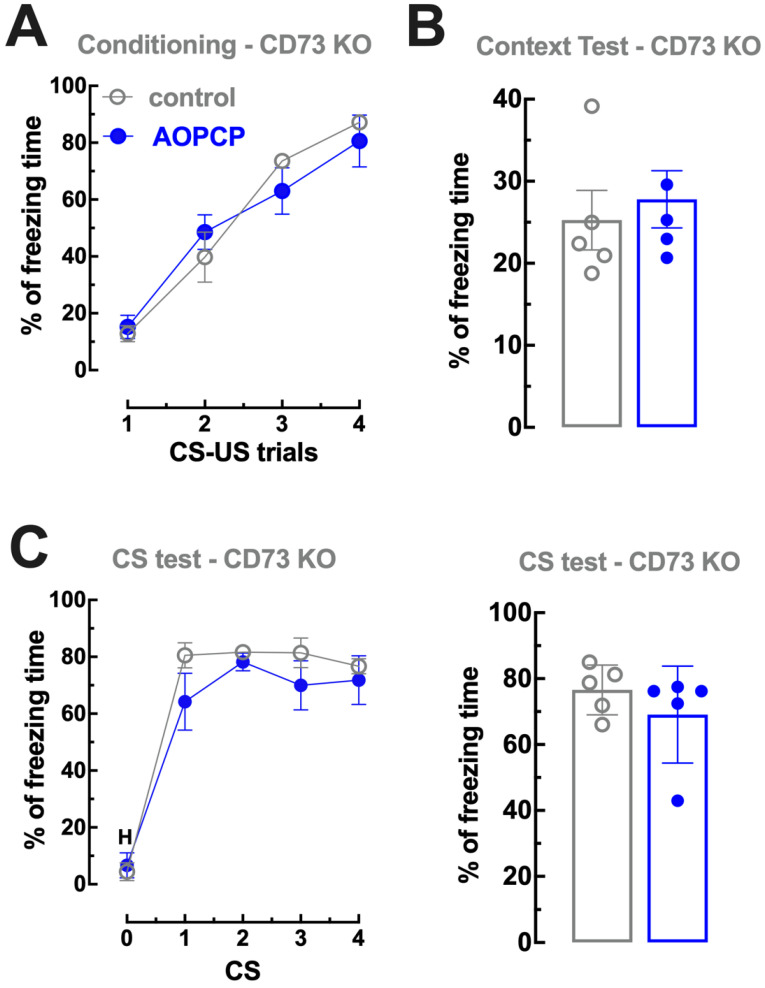
The effect of AOPCP on fear memory is absent in CD73 knockout mice. (**A**) Fear conditioning of AOPCP-treated and saline-treated CD73 knockout (KO) mice. The learning curve of cued fear conditioning was similar between AOPCP- and saline-treated mice lacking CD73; moreover, both groups significantly increased their freezing behavior at each CS–US trial, showing a normal acquisition of fear. (**B**) Contextual fear memory: AOPCP-treated CD73 KO mice displayed similar freezing behavior comparing to the saline-treated group upon re-exposure to the conditioning chamber (context A) in the absence of the US, 1 day after fear conditioning. (**C**) Cued fear memory: AOPCP-treated CD73 KO mice displayed a cued fear memory, assessed as a percentage of freezing, similar to saline-treated WT littermates upon presentation of the CS in the 4 trials after a 2 min habituation (H) period in the absence of the US, in a novel context B, 2 days after fear conditioning. The values are mean ± SEM of n = 5 experiments.

In order to ascertain that the effects of AOPCP were strictly dependent on CD73 inhibition, we tested whether the effects of AOPCP on fear memory were eliminated in CD73-KO mice. The acquisition of fear conditioning was similar in CD73-KO mice treated with saline (n = 3) or with AOPCP (n = 3) (F_3,32_ = 1.036, *p* = 0.399; Figure 3A). Re-exposure to the conditioning context A one day after conditioning induced a similar freezing (t_4_ = 0.503, *p* = 0.629, two tailed unpaired *t*-test) in saline-treated (25.26 ± 3.62% freezing during 12 min, n = 5) or AOPCP-treated mice (27.79 ± 3.49% freezing, n = 5) (Figure 3B). When mice were placed in a novel context B two days after fear conditioning (day 3) and re-exposed to the same CS, vehicle-treated and AOPCP-treated mice showed similar freezing responses upon presentation of the CS (Figure 3C).

**Figure 4:**
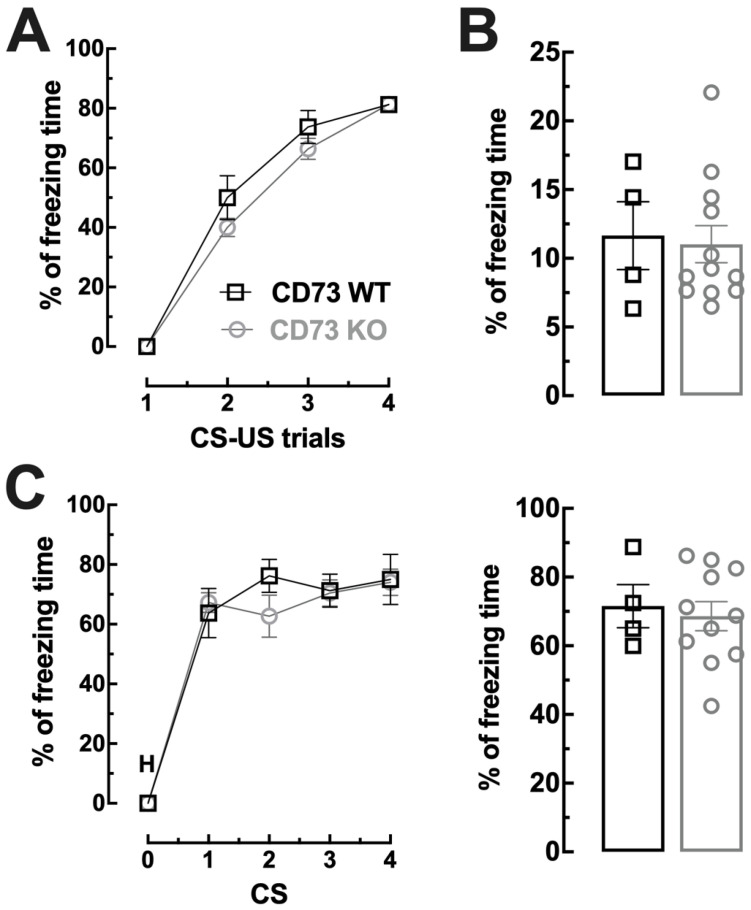
The genetic deletion of CD73 did not modify fear learning and memory. (**A**) Fear conditioning of CD73 KO mice and WT littermates. The learning curve of cued fear conditioning was similar between CD73 KO mice and WT littermates; both groups of mice displayed increased freezing behavior at each CS–US trial, showing a normal acquisition of fear. (**B**) Contextual fear memory: 1 day after fear conditioning, CD73 KO mice and WT littermates were re-exposed to the conditioning chamber (context A) in the absence of the US and displayed similar freezing behavior. (**C**) Cued fear memory: CD73 KO mice and WT littermates had similar freezing behavior upon presentation of the CS (a tone of 80 dB presented during 20 s; 4 trials after 2 min habituation (H) period) in the absence of the US, in a novel context B, 2 days after fear conditioning (graph on the **left**) and an overall equal percentage of freezing throughout the experiment (graph on the **right**). The values are mean ± SEM of n = 7–9 experiments.

Importantly, the impact of AOPCP on context fear conditioning is not recapitulated in the transgenic CD73-KO mice, which display similar acquisition of fear conditioning (F_3,39_ = 1.289, *p* = 0.292), similar context-dependent fear memory (t_14_ = 0.23 *p* = 0.822, two tailed unpaired *t*-test) and similar tone-dependent fear memory (F_4,52_ = 1.143, *p* = 0.347 n = 11) when compared to WT (Figure 4), possibly because of compensatory alterations following the elimination of CD73 since conception.

**Figure 5:**
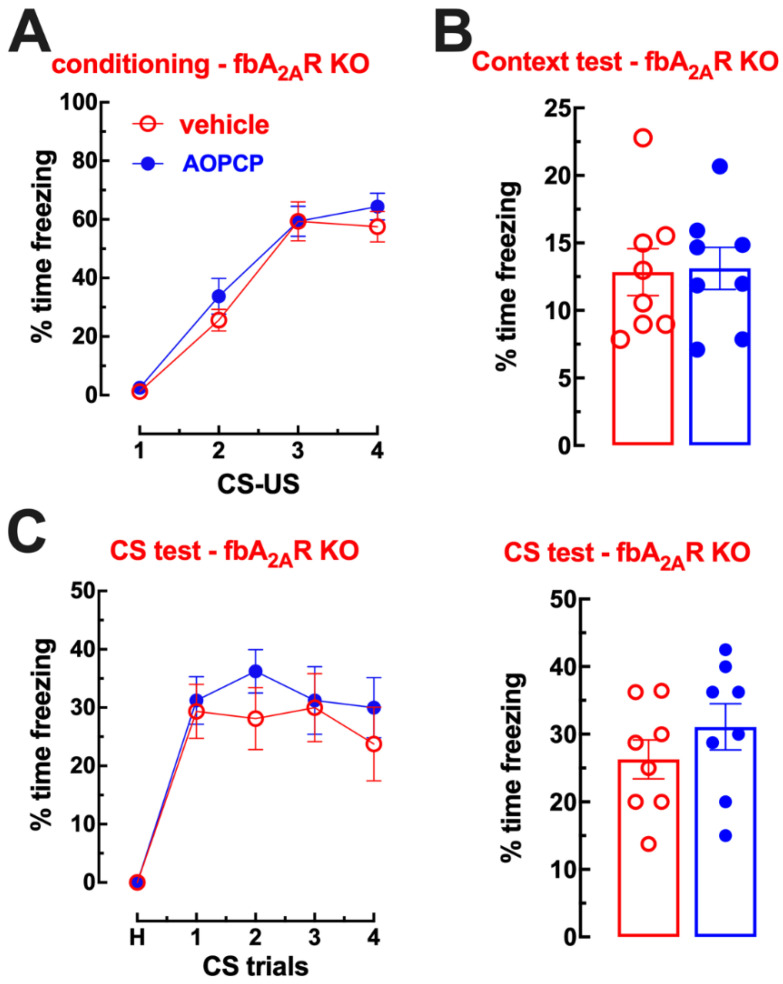
The effect of AOPCP on fear memory is absent in forebrain A_2A_R knockout mice. (**A**) Fear conditioning of AOPCP-treated and saline-treated forebrain (fb)A_2A_R knockout (KO) mice. The learning curve of cued fear conditioning was similar between AOPCP- and saline-treated mice lacking A_2A_R in forebrain neurons; moreover, both groups significantly increased their freezing behavior at each CS–US trial, showing a normal acquisition of fear. (**B**) Contextual fear memory: AOPCP-treated fbA_2A_R KO mice displayed equal freezing behavior comparing to the saline-treated group upon re-exposure to the conditioning chamber (context A) in the absence of the US, 1 day after fear conditioning. (**C**) Cued fear memory: AOPCP-treated fbA_2A_R KO mice displayed a cued fear memory, assessed as a percentage of freezing, similar to the saline-treated WT littermates upon presentation of the CS in the 4 trials after a 2 min habituation (H) period in the absence of the US, in a novel context B, 2 days after fear conditioning. The values are mean ± SEM of n = 8 experiments.

The effects of AOPCP on fear memory essentially phenocopied the effects of the pharmacological or genetic blockade of A_2A_R (see [5]). In order to ascertain that the effects of AOPCP were dependent on A_2A_R function, we tested if the effects of AOPCP on fear memory were eliminated in forebrain neuronal A_2A_R knockout (fbA_2A_R-KO) mice. The acquisition of fear conditioning was similar in fbA_2A_R-KO mice treated with saline (n = 8) or with AOPCP (n = 8) (F_3,56_ = 0.379, *p* = 0.768; Figure 5A). Re-exposure to the conditioning context A one day after conditioning induced a similar freezing (t_4_ = 0.119 *p* = 0.907, two tailed unpaired *t*-test) in saline-treated (12.84 ± 1.75% freezing during 12 min, n = 8) or AOPCP-treated mice (13.12 ± 1.56% freezing, n = 8) (Figure 5B). When mice were exposed to a novel context B two days after fear conditioning (day 3) and re-exposed to the same CS, vehicle-treated and AOPCP-treated fbA_2A_R-KO mice showed similar freezing responses upon presentation of the CS (Figure 5C). As previously described [4], fbA_2A_R-KO mice displayed a lower tone-dependent fear memory (F_4,66_ = 4.546, *p* = 0.003) but a similar acquisition of fear conditioning (F_3,52_ = 0.050, *p* = 0.985) and of context-dependent fear memory (t_14_ = 0.074 *p* = 0.942, two tailed unpaired *t*-test) when compared to WT.

### 2.2. Inhibition of CD73 Blunts A_2A_R-Mediated Control of Amygdala LTP

**Figure 6:**
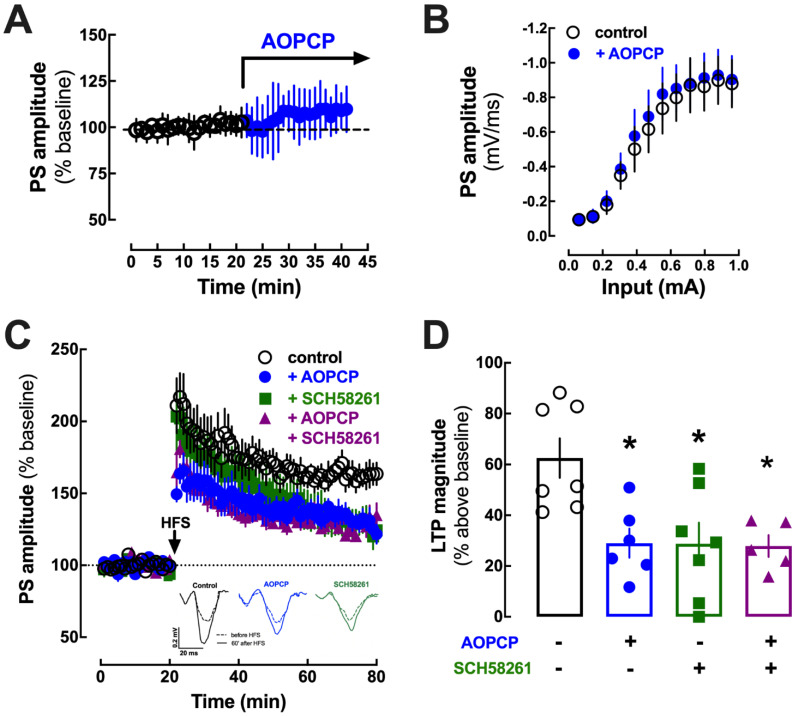
CD73 derived adenosine activates A_2A_R controlling LTP in amygdala slices. The CD73 inhibitor, AOPCP (100 µM), did not significantly affect basal transmission (**A**) or input–output (I/O) curves (**B**) at excitatory synapses between projections of the external capsule (EC) and the lateral amygdala (LA). (**C**,**D**) However, the magnitude of LTP at EC-LA synapses induced by high-frequency stimulation (HFS: 3 × 100 Hz at 5 s intervals) was decreased by AOPCP (100 µM) and by the antagonist of A_2A_R (SCH58261, 50 nM), which were added to the aCSF 20 min before HFS and remained until the end of the experiment; furthermore, there were no synergistic effects between both tested drugs. The values are mean ± SEM of n = 9–10 for baseline and I/O curves and n = 5–7 for LTP experiments; * *p* < 0.05, one-way ANOVA followed by Bonferroni’s post hoc test. The insert in (**C**) shows representative trace recordings of population spikes recorded at EC-LA synapses before HFS (dashed lines) and 60 min after HFS (filled lines) without added drugs (black) or in the presence of either 100 µM AOPCP (blue) or 50 nM SCH58261 (green).

The inhibition of CD73 can lead to an extracellular accumulation of adenine nucleotides [34], potentially unbalancing P2 receptors, affecting synaptic plasticity [35] and amygdala-mediated responses [36] and mood [37]. Thus, the in vitro characterization of the effects of AOPCP on synaptic transmission and plasticity in EC-LA synapses of amygdala slices was carried out in the presence of the generic P2 receptor antagonist PPADS (20 µM). Importantly, PPADS was devoid of effects on LTP in the amygdala (45.7 ± 6.90% over baseline without PPADS and 45.5 ± 13.9% over baseline with PPADS; n = 6; Supplementary Figure S1). AOPCP (100 µM) did not significantly affect basal excitatory synaptic transmission (111.4 ± 4.25% modification of synaptic transmission; n = 10, t_0_ = 2.054, *p* = 0.055 vs. 102.4 ± 1.21%, unpaired *t*-test) in synapses between the external capsule and lateral amygdala (EC-LA) of amygdala slices (Figure 6A); likewise, the input–output curve was also not affected by 100 µM AOPCP (Figure 6B). This excludes an association of ATP-derived adenosine with A_1_R activation, which efficiently controls basal synaptic transmission in the amygdala [38] as heralded by the ability of DPCPX (100 nM) at EC-LA synapses to increase basal synaptic transmission by 30.4 ± 5.51% (n = 9; *p* < 0.001 vs. basal of 0%, *t*-test) while being devoid of effects on the magnitude of LTP (64.3 ± 8.55% over baseline without and 64.7 ± 9.06% with DPCPX; n = 6, *p* = 0.937, *t*-test; Supplementary Figure S2). In contrast, AOPCP decreased LTP magnitude (from 62.6 ± 7.79% over baseline without AOPCP to 29.0 ± 5.69% over baseline with AOPCP; n = 6–7, F_3,21_ = 6.004, *p* = 0.018, one-way ANOVA followed by Bonferroni’s post hoc test) in slices from wild type mice (WT) (Figure 6C,D). This shows that AOPCP selectively affects synaptic plasticity, rather than basal synaptic transmission, in a manner analogous to the impact of the A_2A_R antagonist SCH58261 [5]. In fact, the A_2A_R antagonist SCH58261 (50 nM) phenocopied the effect of AOPCP on amygdala LTP (62.6 ± 7.79% without, 28.8 ± 8.29% with SCH58261, n = 6–7, F_3,21_ = 6.004, *p* = 0.004, one-way ANOVA followed by Bonferroni’s post hoc test; Figure 6C,D). Moreover, AOPCP (100 µM) and SCH58261 (50 nM) did not trigger additive or synergic effects in their simultaneous presence, i.e., the effect of one drug occluded the effect of another on EC-LA LTP (Figure 6C,D), further supporting that CD73-derived adenosine activates A_2A_R controlling LTP in the amygdala.

**Figure 7:**
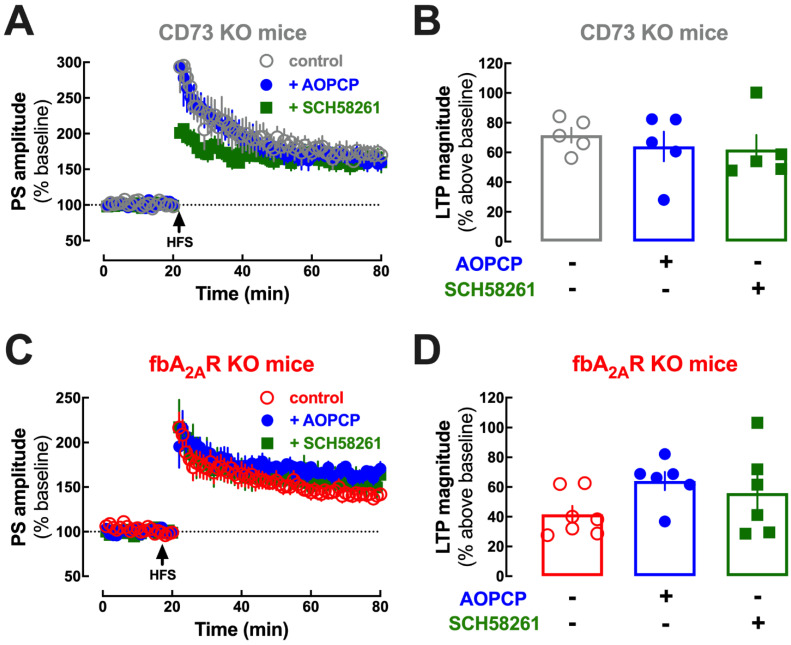
CD73 derived adenosine activates A_2A_R controlling LTP in amygdala slices. The effect of both the CD73 inhibitor, AOPCP (100 µM) and of the A_2A_R antagonist, SCH58261 (50 nM), on the magnitude of long-term potentiation (LTP) induced by high-frequency stimulation (HFS: 3 × 100 Hz at 5 s intervals) at excitatory synapses between projections of the external capsule (EC) and the lateral amygdala (LA) was eliminated in both CD73 KO mice and in forebrain neuronal (fb)A_2A_R KO mice. (**A**) Time course and (**B**) average LTP magnitude in slices from CD73 KO mice, showing that both AOPCP and SCH58261 did not change LTP magnitude. (**C**) Time course and (**D**) bar graph of LTP magnitude in slices from fbA_2A_R KO mice, showing that both AOPCP and SCH58261 did not change LTP magnitude. The values are mean ± SEM of n = 5–7 experiments.

As shown in Figure 7, AOPCP was devoid of effects on LTP magnitude in amygdala slices from CD73-KO mice (n = 5, F_2,12_ = 0.356, *p* = 0.708, one-way ANOVA followed by Bonferroni’s post hoc test; Figure 7A,B), showing that the effect of AOPCP required CD73 activity. Likewise, AOPCP was devoid of effects on LTP magnitude in amygdala slices from fbA_2A_R-KO mice (n = 6–7, F_2,16_ = 2.045, *p* = 0.162, one-way ANOVA followed by Bonferroni’s post hoc test; Figure 7C,D), showing that the effect of AOPCP required A_2A_R activation. Conversely, the effect on EC-LA LTP of the A_2A_R antagonist SCH58261 (50 nM) was eliminated in slices from fbA_2A_R-KO mice (41.6 ± 5.63% without, 56.0 ± 11.80% with SCH58261, n = 6–7, F_2,16_ = 2.045, *p* = 0.669, one-way ANOVA followed by Bonferroni’s post hoc test; Figure 7C,D) and was also abrogated in slices from CD73-KO mice (71.6 ± 4.99% without and 61.9 ± 9.78% with SCH58261, n = 6–7, F_2,16_ = 1.827, *p* = 0.699, one-way ANOVA followed by Bonferroni’s post hoc test; Figure 7A,B). Overall, this shows that that CD73-derived adenosine is strictly required for the A_2A_R-mediated control of amygdala LTP. Importantly, AOPCP and SCH58261 decreased LTP magnitude in amygdala slices from both CD73 and fbA_2A_R WT littermates (Supplementary Figure S3) as observed in WT mice (Figure 6C,D).

It is worth noting that the similar impact on LTP of the pharmacological inhibition of CD73 and of the blockade of A_2A_R is not recapitulated in the transgenic CD73-KO mice, but it is in fbA_2A_R-KO mice. Indeed, fbA_2A_R-KO display decreased LTP magnitude compared to wild type animals (62.6 ± 7.79% over baseline in WT vs. 41.6 ± 5.63% over baseline in fbA_2A_R -KO; n = 7, t_0_ = 2.180, *p* = 0.049, unpaired *t*-test), unlike CD73-KO mice (62.6 ± 7.79% over baseline in WT vs. 63.6 ± 8.99% over baseline in CD73-KO; n = 6–7, t_0_ = 0.088, *p* = 0.932, unpaired *t*-test), possibly because the genetic deletion of A_2A_R in fbA_2A_R-KO mice is conditioned by the expression of the CAMKIIα promoter [39,40], which increases only after the second postnatal week, whereas CD73-KO mice lacked CD73 protein since conception, which likely led to compensatory alterations.

## 3. Discussion

The present study confirms the role of neuronal adenosine A_2A_ receptors (A_2A_R) in the control of fear memory and identifies CD73 as the source of extracellular adenosine responsible for the activation of these A_2A_R controlling fear memory. Accordingly, the control by A_2A_R of LTP magnitude in synapses of excitatory cortico-thalamic afferents into the amygdala was also identified to be dependent on CD73 activity to form the pool of extracellular adenosine ensuring the activation of A_2A_R at excitatory synapses of the lateral amygdala [5].

We previously showed that A_2A_R control fear memory and the underlying process of synaptic plasticity in the amygdala [5]. We now report that the inhibition with AOPCP of the CD73-mediated formation of extracellular adenosine phenocopies the effect of the selective inhibition of A_2A_R with SCH58261 on fear memory and amygdala LTP. Furthermore, we observed that the effects of AOPCP on fear memory and the effects of AOPCP and of SCH58261 on amygdala LTP are eliminated in CD73-KO mice and in fbA_2A_R-KO mice, re-enforcing the association of CD73-mediated formation of extracellular adenosine and the selective activation of A_2A_R to control fear memory and the underlying LTP in amygdala synapses receiving sensory information from the external capsule. This prompts the conclusion that ATP-derived extracellular adenosine is the preferential pathway responsible for the activation of A_2A_R in the amygdala to control fear memory, in a manner analogous to that previously described in other brain circuits, namely of the hippocampus controlling memory deterioration [25] or of the striatum controlling motor dysfunction [32]. However, it remains to be investigated what might be the source of extracellular ATP and if the pattern of ATP release in the amygdala is similar to that reported in other brain regions, such as the hippocampus [27,28]. The clarification of the spatiotemporal pattern of extracellular ATP levels in amygdala excitatory synapses will also be paramount to understand if the main purpose of synaptically released ATP is to serve as a source of extracellular adenosine to activate A_2A_R or if extracellular ATP might fulfill additional roles signaling through P2 receptors (reviewed in [22]), which have not yet been investigated in amygdala circuits, although affecting amygdala-mediated responses [36].

Previous pharmacological manipulations of CD73 have not detected effects of AOPCP in the brain-associated behavior outputs of normal animals [24,32,41,42]. In contrast, AOPCP mostly attenuated behavioral deficits caused by noxious stimuli to the brain [32,42,43,44,45]. Likewise, CD73-KO mice have no major brain-related phenotype unless subjected to stressful situations [24,25,32,33,46,47]. Overall, these observations are in agreement with the concept that extracellular ATP is a danger signal in the brain [48], and thus, a role of CD73 can only be made evident in stressful conditions in which there is sufficient ATP release to increase the activity of the ecto-nucleotidase pathway. Curiously, AOPCP controlled the expression of fear memories, suggesting that CD73-mediated formation of extracellular adenosine might have a particular relevance in the control of the processing of emotional memories when compared to other types of memory processing, such as spatial reference memory, which is only affected by AOPCP when perturbed by noxious stimuli [25]. Thus, this different impact of CD73-mediated formation of extracellular adenosine may offer potential therapeutic opportunities to target CD73 to selectively manipulate emotional memory processing.

The control by A_2A_R of the processing of fear stimuli likely involves an impact of A_2A_R in different brain regions [4]. This is most evident in the acquisition of fear conditioning, where selective A_2A_R antagonists are devoid of effects, whereas the downregulation of A_2A_R selectively in the amygdala attenuates the acquisition of fear learning. We now observed that the inhibition of CD73-mediated formation of extracellular adenosine mimicked the lack of effect of SCH58261 on the acquisition of fear conditioning; this implies that the association of CD73-mediated formation of extracellular adenosine with the activation of A_2A_R does not occur selectively in the amygdala but might be widespread though circuits in different brain areas. Indeed, previous studies have described a similar tight association between CD73 activity and the activation of A_2A_R during neurodevelopment [49], in circuits of the dorsal striatum [32,50] and of the normal [25] or diseased hippocampus [26,51], supported by the physical association of A_2A_R and CD73 in the striatum [24]. A similar association of CD73 and A_2A_R was concluded in other cells or tissues such as neuromuscular junction [52], astrocytes [53], “activated” microglia [54,55], neutrophils [56], B cell lymphocytes [57], T cell lymphocytes [58], fibroblasts [59], cardiac valve interstitial cells [60] or mesenchymal stem cells [61]. Overall, this evidence supports a general association of CD73-mediated formation of extracellular adenosine with the activation of A_2A_R, although an association of CD73 activity with A_2B_R has been described in some tissues/cells where A_2B_R-mediated responses are predominant, such as osteoblasts [62], dendritic cells [63] or glioblastoma cells [64].

In conclusion, the presently identified pathway of CD73 activity as a source of extracellular adenosine sustaining the activation of A_2A_R to control amygdala LTP and fear memory prompts consideration of the manipulation of CD73 as a new possibility to pharmacologically correct different behavioral responses that are proposed to be dependent on the function of amygdala A_2A_R, in particular, abnormal fear memory processing [5], social interactions [8], anxiety [6] or binge eating [65].

## 4. Materials and Methods

### 4.1. Animals

CD73-knockout (KO) mice and forebrain neuronal (fb)A_2A_R-KO mice were generated in a C57bl\6 background and crossbred as previously described [24,25]. Male C57BL/6 mice (Charles River, Barcelona, Spain), KOs (CD73-KO and fbA_2A_R-KO) and wild type littermates of 8–12 weeks old were used in the experiments. Mice were kept in groups of three to four per cage in a temperature-controlled room (22 ± 1 °C), with a 12 h light/12 h dark cycle (lights on at 7:00 AM) and with free access to food and water. All manipulations followed the principles and procedures outlined as “3 Rs” in the EU guidelines (210/63) and were approved by the Animal Care Committee of the Center for Neuroscience and Cell Biology (ORBEA 238_2019/14102019).

### 4.2. Intracerebroventricular Drug Administration

The animals were anaesthetized with avertin (1.3% tribromoethanol, 0.8% tert-amyl alcohol) and placed in a stereotactic apparatus (Stoelting, Wood Dale, IL, USA). Then, mice were subjected to icv cannulation (x = −0.22 mm; y = −1 mm; z = −2.25 mm). This procedure consisted of the application of a cannula (Plastics One, Roanoke, VA, USA), which allowed us to administrate/infuse 1 nmol AOPCP (at a rate of 1 μL/min), reaching each of the lateral ventricles of the brain; AOPCP was bilaterally administered three different times (at 24 h intervals) before (the third time was 1 h before starting the acquisition in fear conditioning protocol) and after (the first time was administrated immediately after the acquisition) the start of the fear conditioning protocol (see Figure 1).

### 4.3. Behavioral Analysis—Fear Conditioning

Behavioral tests were conducted between 9:00 AM and 1:00 PM (light phase) in a sound attenuated room with 15 lux illumination, where the mice had been habituated for 1 h before beginning the tests. The apparatus and objects were cleaned with a 70% alcohol solution and rinsed with water after each session. The behavior was video-monitored with ANY-mazeTM (Stoelting, Wood Dale, IL, USA). Locomotion was evaluated in an open-field arena, measuring the distance travelled during a 10 min period, as previously described [7].

Fear conditioning was performed as previously described [5]. Mice were first placed in context A with four successive presentations of an auditory conditioned stimulus (CS; 80 dB for 20 s at 4 kHz) paired with a foot-shock unconditioned stimulus (US; 0.5 mA for 2 s, delivered 20 s after the beginning of CS) and with a 120 s inter-trial interval, for a total time of 12 min (acquisition session). At day 2, mice were returned to context A to test their contextual freezing behavior for 12 min. At day 3, mice were placed in a completely different chamber (different environment and lux conditions; context B), the CS was presented as it was previously in context A and the freezing behavior was measured for 12 min. Thus, we evaluated two types of conditioned fear: contextual fear conditioning and cued fear conditioning through the analysis of the time spent freezing, defined as “absence of movement except for respiration”. The outline of this fear conditioning protocol is presented in Figure 1.

### 4.4. Electrophysiology

Electrophysiological recordings were carried out as previously described [5]. Briefly, each mouse was killed by decapitation and the brain was quickly removed and placed in ice-cold modified artificial cerebrospinal fluid (aCSF) containing (in mM); 124 NaCl, 4.5 KCl, 2 CaCl_2_, 1 MgCl_2_, 26 NaHCO_3_, 1.2 NaH_2_PO_4_ and 10 D-glucose, bubbled with a gas mixture of 95% O_2_ and 5% CO_2_. The brain was then sectioned into 400 µm thick horizontal slices (cut from the ventral towards the dorsal part of the brain) with a vibratome (1500, Leica, Wetzlar, Germany). Brain slices were allowed to equilibrate in gassed aCSF for 1 h kept at 32 °C and another 40 min at room temperature before being transferred to a submerged recording chamber and continuously superfused with gassed aCSF (3 mL/min) kept at 30 ± 1 °C. Visual control through a magnifier (World Precision Instruments, Hertfordshire, UK) allowed the correct placement of the electrodes. Extracellular field recordings were obtained using borosilicate micropipettes filled with a 4 M NaCl solution (2–4 MΩ), placed at the lateral amygdala (LA), and test stimuli were delivered via a S44 stimulator (Grass Instruments, West Warwick, RI, USA) at a frequency of 0.05 Hz through a bipolar twisted tungsten wire placed at the external capsule (EC). The population spike (PS) response was used to estimate synaptic efficacy, and its amplitude was measured as the distance from the maximal negative peak to a line tangent to the lower and upper shoulders of the PS. Recordings were obtained with an ISO-80 amplifier (World Precision Instruments, Hertfordshire, UK) and digitized using an ADC-42 board (Pico Technologies, Pelham, NY, USA). Averages of three consecutive responses were continuously monitored on a PC-type computer using the WinLTP 1.01 software [66].

To evaluate basal neurotransmission, input–output curves (I/O) were first acquired by continuously increasing the current applied to the EC fibers and recording the amplitude of the evoked PS response at the LA, starting with a current which elicited no response and terminating when the evoked PS amplitude stabilized at a maximal amplitude. The I/O curve allowed choosing a stimulus that evoked a signal of circa 40% of the maximal PS amplitude. The effect of drugs, added through the aCSF, on baseline was calculated as the percentage of change of the PS amplitude in the presence versus absence of the drug by comparing the average of the signal in the last 5 min in the presence of the drug (after the signal plateaued) with the average of the signal in the last 5 min in the absence of the drug.

LTP was induced by high frequency stimulation (HFS) consisting of three trains of pulses of 100 Hz delivered with a 5 s inter-train interval. The magnitude of LTP was calculated by comparing the average of PS amplitudes 50–60 min after HFS with the average of the PS amplitudes 10 min before the HFS (baseline). Drugs were added to the aCSF 20 min before the application of the LTP protocols and maintained until the end of the experiments. The effect of drugs on LTP was assessed by comparing the magnitude of LTP in the absence and presence of the drug in experiments carried out in different slices from the same animal. The values are presented as mean ± SEM of n (number of animals) experiments.

### 4.5. Drugs

For the in vivo studies, adenosine 5′-(α,β-methylene)diphosphate (AOPCP, selective inhibitor of CD73; Sigma-Aldrich, Sintra, Portugal) was made up to a 10 mM stock solution in saline and used at a supra-maximal and selective dose of 1 nmol/µL [43]. For the in vitro studies, the following drugs were used in previously defined supramaximal and selective concentrations: AOPCP (100 µM; [25]), pyridoxalphosphate-6-azophenyl-2′,4′-disulfonic acid tetrasodium salt (PPADS, 20 µM, non-selective antagonist of ATP P2 receptors; [67]), 7-(2-phenylethyl)-5-amino-2-(2-furyl)-pyrazolo-[4,3-e]-1,2,4-triazolo [1,5-c]pyrimidine (SCH58261, 50 nM; A_2A_R selective antagonist; [23]), 4-[2-[[6-amino-9-(*N*-ethyl-β-D-ribofuranuronamidosyl)-9*H*-purin-2-yl]amino]ethyl]benzenepropanoic acid hydrochloride (CGS21680, 30 nM; A_2A_R selective agonist; [68]), 8-cyclopentyl-1,3-dipropylxanthine (DPCPX, 100 nM; A_1_R selective antagonist; [69]) and N^6^-cyclopentyladenosine (CPA, 100 nM; A_1_R selective agonist; [70]). PPADS, SCH58261, CGS21680, DPCPX and CPA were purchased from Tocris Bioscience (Biogen Cientifica, Madrid, Spain) and AOPCP from Sigma-Aldrich (Sintra, Portugal). DPCPX, CPA, SCH58261 and CGS21680 were first prepared as stock solutions in dimethylsulfoxide (Sigma-Aldrich, Sintra, Portugal) and then were diluted to the final concentration in aCSF; the stock solutions of AOPCP and PPADS were prepared in milliQ water and then diluted in aCSF to their final concentration.

### 4.6. Statistical Analysis

Each analyzed parameter was estimated based on experiments carried out in four or more animals, and the individual sample size (n = number of animals) is specified for each experiment. All data are displayed as mean ± SEM and significance was considered with 95% confidence using either a one-sample *t* test to assess the effect of any individual drug or treatment in electrophysiological experiments, a two-way Student’s *t* test with Welsh correction for comparison between two groups and one-way ANOVA (followed by a Bonferroni’s post hoc test) or two-way ANOVA (followed by a Newman–Keuls post hoc test) to compare multiple groups.

## Data Availability

The data supporting the findings of this study are available from the corresponding author upon reasonable request.

## Supplementary Materials

The supporting information can be downloaded at: https://www.mdpi.com/article/10.3390/ijms232112826/s1.

## Abbreviations

A_2A_R, A_2A_ receptor; aCSF, artificial cerebrospinal fluid; AOPCP, αβ-methylene ADP; CS, conditional stimulus; EC, external capsula; DPCPX, 1,3-dipropyl-8-cyclopentylxanthine; fbA_2A_R, forebrain neuronal A_2A_ receptor; HFS, high-frequency stimulation; I/O, input/output; KO, knockout; LA, lateral amygdala; LTP, long-term potentiation; PPADS, pyridoxalphosphate-6-azophenyl-2′,4′-disulfonic acid tetrasodium salt; SCH58261, 7-(2-phenylethyl)-5-amino-2-(2-furyl)-pyrazolo-[4,3-e]-1,2,4-triazolo [1,5-c]pyrimidine; US, unconditional stimulus; WT, wild-type.
