# Supplementary material for: CD73-Mediated Formation of Extracellular Adenosine Is Responsible for Adenosine A2A Receptor-Mediated Control of Fear Memory and Amygdala Plasticity
Source: Int J Mol Sci. 2022 Oct 24;23(21):12826. doi: 10.3390/ijms232112826 (PMC9653840; doi:10.3390/ijms232112826)
Supplement: Supplementary file 1 [file ijms-23-12826-s001.zip › ijms-1940509-supplementary.pdf]

Supplementary Data

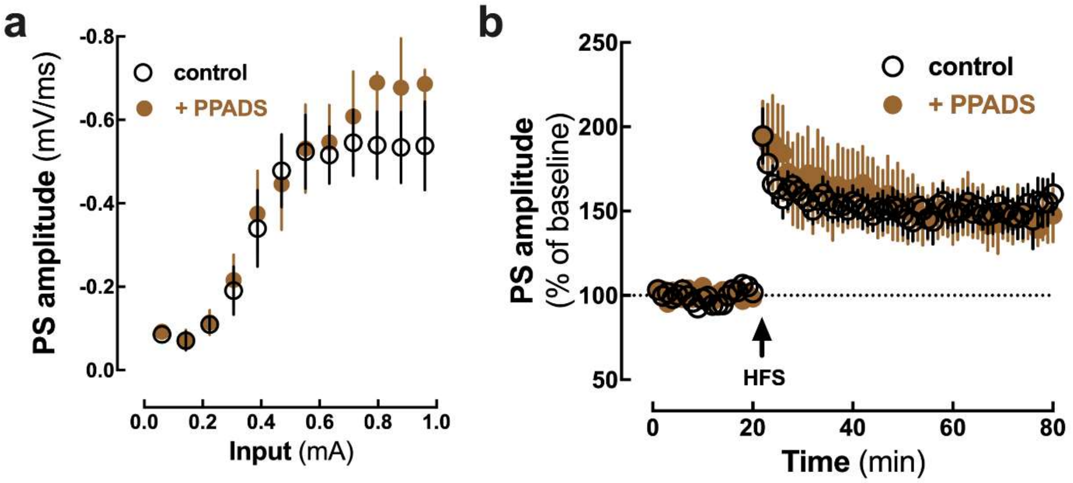

Supplementary Figure S1. Blockade of ATP P2 receptors does not affect basal transmission nor LTP in the amygdala. (**a**) Superimposable input-output (I/O) curves at excitatory synapse between projection of the external capsule (EC) and the lateral amygdala (LA) in the absence and presence of the generic antagonist of P2 receptors, PPADS (20 μM); (**b**) Time course of LTP in mouse slices: LTP was consistently induced at EC-LA pathway by high frequency stimulation (HFS, 3 × 100 Hz at 5 s intervals) in the absence and presence of PPADS (20 μM); PPADS was added 20 min before HFS and did not alter LTP magnitude. The values are mean ± SEM; n = 7–9.

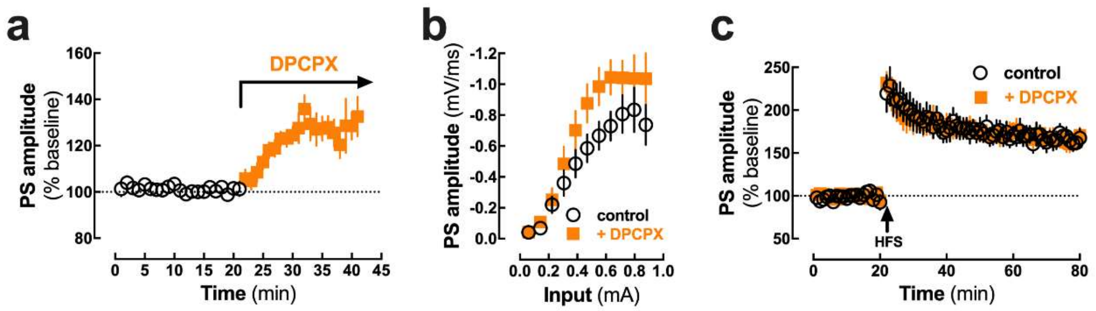

Supplementary Figure S2. Adenosine A1 receptors tonically inhibit basal transmission but do not affect LTP in the amygdala. (**a**) The selective antagonist of A1 receptors, DPCPX (100 nM) increased basal transmission (**a**) as well as input-output (I/O) curves (**b**) at excitatory synapse between projection of the external capsule (EC) and the lateral amygdala (LA). (**c**) In contrast, the magnitude of LTP at EC-LA synapses induced by high-frequency stimulation (HFS: 3 × 100 Hz at 5 s intervals) was not affected by DPCPX (100 nM). The values are mean ± SEM; n = 6–10.

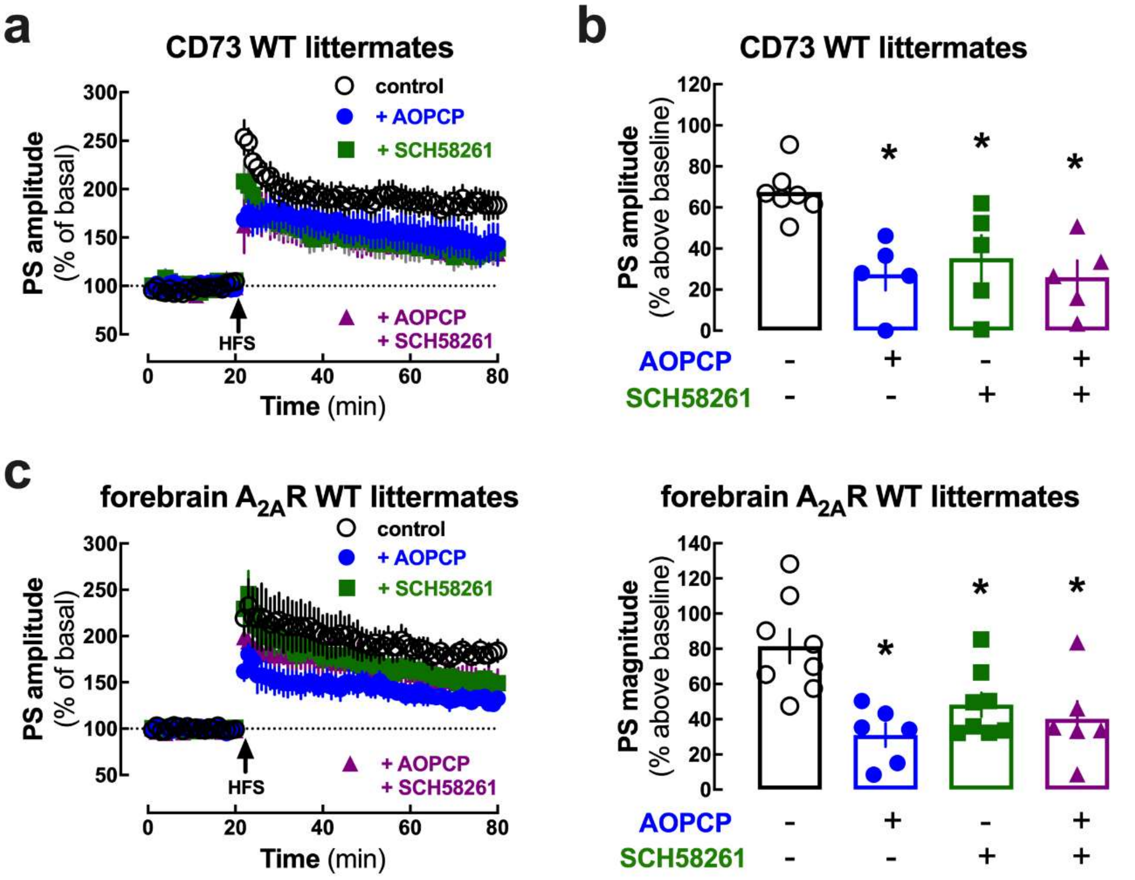

Supplementary Figure S3. Inhibition of CD73 and blockade of $A_{2A}R$ similarly decreased LTP magnitude in amygdala slices from WT littermates of CD73 KO and fb$A_{2A}R$ KO mice. LTP was consistently induced at excitatory synapse between projection of the external capsule (EC) and the lateral amygdala (LA) by high frequency stimulation (HFS: 3 × 100 Hz at 5 s intervals) in all experimental groups. (**a**) Time course of LTP and (**b**) average magnitude of LTP in slices from CD73 WT littermate mice; the CD73 inhibitor AOPCP (100 μM) and the $A_{2A}R$ antagonist SCH58261 (50 nM), added 20 min before HFS, decreased LTP magnitude to similar levels and did not have a synergistic effect. (**c**) Time course of LTP and (**d**) average magnitude of LTP in slices from forebrain $A_{2A}R$ WT littermate mice; AOPCP (100 μM) and SCH58261 (50 nM), added 20 min before HFS, decreased LTP magnitude to similar levels and did not have

a synergistic effect. The values are mean ± SEM, n = 5–8; * $p < 0.05$ one-way ANOVA followed by Bonferroni's post hoc test.
